# Correction: IgM antibodies derived from memory B cells are potent cross-variant neutralizers of SARS-CoV-2

**DOI:** 10.1084/jem.2022084908172022c

**Published:** 2022-08-29

**Authors:** Malika Hale, Jason Netland, Yu Chen, Christopher D. Thouvenel, Katherine Nabel Smith, Lucille M. Rich, Elizabeth R. Vanderwall, Marcos C. Miranda, Julie Eggenberger, Linhui Hao, Michael J. Watson, Charles C. Mundorff, Lauren B. Rodda, Neil P. King, Miklos Guttman, Michael Gale, Jonathan Abraham, Jason S. Debley, Marion Pepper, David J. Rawlings

Vol. 219, No. 9 | 10.1084/jem.20220849 | August 8, 2022

The authors regret that in the original version of their article, the relative affinity graphs for 204 IgM and IgG in Fig. 2 D were accidentally reversed while preparing figures for final publication. The remainder of the figure, including the Kd reported in the associated table, is correct as originally published. The corrected Fig. 2 D is shown here.

**Figure 2.**
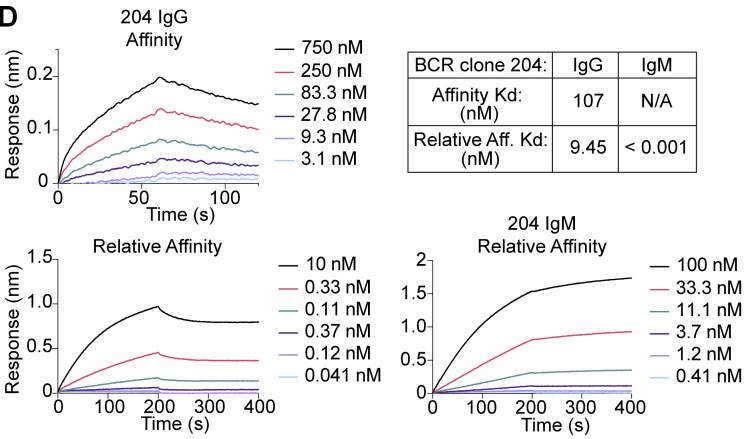


The error appears in PDFs downloaded before August 18, 2022.

